# Developmental delays in cortical auditory temporal processing in a mouse model of Fragile X syndrome

**DOI:** 10.1186/s11689-023-09496-8

**Published:** 2023-07-29

**Authors:** Katilynne Croom, Jeffrey A. Rumschlag, Michael A. Erickson, Devin K. Binder, Khaleel A. Razak

**Affiliations:** 1 Graduate Neuroscience Program, University of California, Riverside, USA; 2 Department of Otolaryngology-Head and Neck Surgery, Medical University of South Carolina, Charleston, USA; 3 Department of Psychology, University of California, Riverside, USA; 4 Biomedical Sciences, School of Medicine, University of California, Riverside, USA

**Keywords:** Autism spectrum disorders, Fragile X syndrome, Speech processing, Temporal processing, Sensory hypersensitivity, Language, Neurodevelopment, Cerebral cortex

## Abstract

**Background:**

Autism spectrum disorders (ASD) encompass a wide array of debilitating symptoms, including sensory dysfunction and delayed language development. Auditory temporal processing is crucial for speech perception and language development. Abnormal development of temporal processing may account for the language impairments associated with ASD. Very little is known about the development of temporal processing in any animal model of ASD.

**Methods:**

In the current study, we quantify auditory temporal processing throughout development in the *Fmr1* knock-out (KO) mouse model of Fragile X Syndrome (FXS), a leading genetic cause of intellectual disability and ASD-associated behaviors. Using epidural electrodes in awake and freely moving wildtype (WT) and KO mice, we recorded auditory event related potentials (ERP) and auditory temporal processing with a gap-in-noise auditory steady state response (gap-ASSR) paradigm. Mice were recorded at three different ages in a cross sectional design: postnatal (p)21, p30 and p60. Recordings were obtained from both auditory and frontal cortices. The gap-ASSR requires underlying neural generators to synchronize responses to gaps of different widths embedded in noise, providing an objective measure of temporal processing across genotypes and age groups.

**Results:**

We present evidence that the frontal, but not auditory, cortex shows significant temporal processing deficits at p21 and p30, with poor ability to phase lock to rapid gaps in noise. Temporal processing was similar in both genotypes in adult mice. ERP amplitudes were larger in *Fmr1* KO mice in both auditory and frontal cortex, consistent with ERP data in humans with FXS.

**Conclusions:**

These data indicate cortical region-specific delays in temporal processing development in *Fmr1* KO mice. Developmental delays in the ability of frontal cortex to follow rapid changes in sounds may shape language delays in FXS, and more broadly in ASD.

## Background/Introduction

Auditory temporal and spectral modulation cues shape speech recognition [1, 2].  The ability to discriminate temporal fine structure is critical for speech processing [3], and the ability to encode subtle differences in temporal modulations is present from a very young age in humans [4]. The inability of the auditory system to process rapidly changing acoustic input during development may disrupt perception of speech, phonological processing and cause language impairments [5]. Temporal processing acuity in infancy predicts language development in ~ 2 yr old children [6]. Abnormal sensory processing and language development is consistently reported in children with autism spectrum disorders (ASD) [7–11]. Individuals with ASD show deficits in detection of sound duration, onset and offset, and rapid changes in spectrotemporal properties [12–16]. Children with ASD show difficulties reproducing the lengths of auditory stimuli, and both children and adults with ASD produce abnormal neural responses to fluctuations in pitch of repeated, sequential auditory stimuli [17–19]. Increased gap-detection thresholds, a paradigm commonly used to assess auditory temporal processing, are seen in humans with ASD. Notably, impaired gap detection thresholds in children were associated with lower phonological processing scores [8]. These studies in humans provide support for the hypothesis that auditory temporal processing deficits may shape abnormal speech and language function in ASD. A link between abnormal temporal processing and developmental dyslexia has also been proposed, suggesting broader consequences in development [20].

While speech and language function cannot be directly studied in animal models, temporal processing can be quantified. However, the developmental trajectory and underlying neural mechanisms of temporal processing deficits in neurodevelopmental disorders remain unclear and would require a translation-relevant animal model. Identifying when temporal processing deficits arise is critical for determining optimal treatment windows for potential therapeutic tests in pre-clinical models and in clinical studies. Here we present a novel method to assess rapid gap-in-noise temporal processing using EEG recordings in an ASD model mouse, which can be translated relatively easily to humans, and we show robust cortical region-specific developmental delays in auditory temporal processing.

Fragile X syndrome (FXS) is a leading cause of inherited intellectual deficits and ASD-associated behaviors such as repetitive behaviors, sensory, cognitive and social impairments [21–25]. Humans with FXS show speech deficits and language impairments [7, 26–29]. FXS affects up to 1 in 4000/7000 male/female individuals, respectively, and results from the silencing of the Fragile X Messenger Ribonucleoprotein (*Fmr1*) gene on the X chromosome [30, 31]. This leads to a partial or complete loss of the Fragile X Messenger Ribonucleoprotein (FMRP) and consequent alterations in synaptic development and plasticity in the brain [32, 33]. Clinical, behavioral and electrophysiological studies have demonstrated sensory hypersensitivity in humans with FXS across multiple domains [25, 34–38].

Sensory hypersensitivity is also consistently seen in the *Fmr1* KO mouse model of FXS [39–42]. Notably, *Fmr1* KO mice display abnormal responses to auditory stimuli similar to humans, providing a translational platform to study developmental profiles and neural mechanisms of sensory circuit pathophysiology [43]. EEG recordings from humans with FXS show altered cortical oscillatory activity that may result in sensory hypersensitivity [44]. More specifically, increased broadband gamma frequency power was seen in humans with FXS compared to healthy controls [31]. When time-varying auditory stimuli were used, there was a deficit in narrowband (~ 40 Hz) evoked gamma synchronization. There is also enhanced amplitude and reduced habituation of auditory ERPs in humans with FXS [45]. These results suggest elevated baseline cortical activity in FXS that disrupts the ability of cortical generators to synchronize their oscillations to dynamic stimuli. Enhanced responses to repeated stimuli also indicate elevated ongoing cortical activity. Similar EEG phenotypes are seen in the *Fmr1* KO mice: elevated broadband gamma power, reduced narrowband gamma synchronization and increased cortical responses to repeated stimuli have been identified in *Fmr1* KO mice [42, 46–49]. Taken together, the similarities in sensory hypersensitivity behaviors and in EEG phenotypes across humans and mice indicates that the *Fmr1* KO mouse is a useful model to address sensory dysfunction in FXS.

Developmental abnormalities in cell size and expression of synaptic markers are seen in the auditory brainstem of the *Fmr1* KO mice, a region strongly implicated in high resolution temporal processing [50]. This suggests that auditory temporal processing abnormalities may emerge early in development. However, the development of temporal processing has not been studied in the *Fmr1* KO mice, or indeed in any animal model of ASD. The current study tested the hypothesis that cortical temporal processing and auditory sensitivity deficits are present in the *Fmr1* KO mice from early development. We recorded EEG signals from both the auditory and frontal cortex (AC, FC) in *Fmr1* KO and wild-type (WT) mice at three ages: p21, p30 and p60. To quantify temporal processing fidelity, we utilized a 40 Hz gap-in-noise ASSR (auditory steady state response, henceforth, gap-ASSR) paradigm to assess the cortex’s ability to consistently respond to brief gaps in noise at varying modulation depths [51]. Gap stimuli have been used widely in both humans and mice to study auditory temporal acuity, and EEG recordings can be conducted in humans and mice relatively more easily than single unit recordings [52, 53]. Children with autism show reduced ability to integrate information present in temporal gaps in background sound, providing additional rationale to use gaps-in-noise stimuli to evaluate temporal processing [54]. Regional differences in cortical phenotypes are present in *Fmr1* KO mice. In particular, multiple mouse model studies suggest auditory temporal processing abnormalities may be larger in the FC than AC. For example, Wieczerzak et al. reported reduced recovery of ASSR in FC, compared to AC, following noise induced hearing loss [55]. Lovelace et al. reported a deficit in ASSR in the FC, but not AC, of the adult *Fmr1* KO mouse [56]. Temporal processing impairments are seen in an auto-immune disorder mouse model with neocortical ectopias in the FC [57]. It is not known if these regional differences in any mouse model are present across development, or only at specific ages. Therefore, we compared FC and AC in terms of temporal processing across 3 different ages. Additionally, we assessed the response magnitude of auditory ERP, as they are well characterized in humans with FXS and consistently show enhanced amplitudes. We hypothesized that *Fmr1* KO mice would show a deficit in auditory temporal processing and increased ERP amplitudes compared to WT in both the AC and FC across all 3 developmental time points.

## Methods

### Mice

All procedures were approved by the Institutional Animal Care and Use Committee at the University of California, Riverside. Mice were obtained from an in-house breeding colony that originated from Jackson Laboratory (Bar Harbor, ME). The mice used for the study are sighted FVB wild-type (Jax, stock# 004828; WT) and sighted FVB *Fmr1* knock-out (Jax, stock# 004624; *Fmr1* KO). The FVB background strain was chosen (and not C57bl6/J) because our prior developmental work examining cortical parvalbumin and perineuronal nets as well as single unit responses in the auditory cortex and the inferior colliculus have used the FVB strain [46, 58]. Significant developmental deficits were observed in *Fmr1* KO mice in the FVB strain of mice, predicting temporal processing deficits.

One to five mice were housed in each cage under a 12:12-h light–dark cycle and fed ad libitum. A cross-sectional, as opposed to a longitudinal, design was used in this study as it is impractical to place epidural screw electrodes in brains and skulls that are still developing. The following age ranges and sample sizes were used in this study: WT [p21 (*n* = 10), p30 (*n* = 10), p60 (*n* = 11)] and *Fmr1* KO [p21 (*n* = 10), p30 (*n* = 10), p60 (*n* = 11)]. The ages were selected for this study based on previous findings. Decreased PNN expression surrounding parvalbumin-positive interneurons and cortical hyperexcitability are observed in *Fmr1* KO mice at p21 [59]. Additionally, the p14-21 age corresponds to the critical period for responses to tones and maturation of tonotopic maps in the auditory cortex [60, 61]. P30 was chosen because response selectivity to complex sounds has not matured in the auditory cortex until this age [62]. We chose p60 age group to represent young adulthood. Only male mice were studied.

### Surgery

Different groups of mice underwent epidural electrode implant surgery at three developmental timepoints: p18-20, p27-p29, p57-p66. Mice were anesthetized using intraperitoneal (i.p.) injections of either 80/20 mg/kg of ketamine/xylazine (young mice) or 80/10/1 mg/kg ketamine/xylazine/acepromazine (adult mice). The anesthetic state was monitored closely throughout the procedure by toe pinch reflex every 10–15 min. Ketamine supplements were given if necessary. ETHIQA-XR (1-shot buprenorphine, 3.25 mg/kg body weight) was administered via subcutaneous injection prior to surgery. Following the removal of fur and skin, and sterilization (alcohol and iodine wipes) of the scalp, an incision was made to expose the scalp. A Foredom dental drill was used to drill ~ 1 mm diameter holes in the skull over the right AC, right FC, and left occipital cortex. The screw positions were determined using skull landmarks and coordinates previously reported [48, 51, 56, 63] and were based on single unit mapping [42, 48, 51, 56, 63, 64]. The wires extending from three-channel posts were wrapped around 1 mm screws and driven into the pre-drilled holes. Dental cement was applied around the screws, on the base of the post, and exposed skull, to secure the implant. Mice were placed on a heating pad until fully awake and were allowed 48–72 h for recovery before EEG recordings were made.

### EEG recordings

All EEG recordings were obtained from awake and freely moving mice. EEG recordings were performed at three developmental time points: p20-23, p29-31, p59-p70, which we refer to as p21, p30 and p60, respectively. Recordings were obtained from the AC and FC electrodes, using the occipital screw as reference. Mice were placed in an arena where they could move freely during the recording. The arena was inside a Faraday cage placed on a vibration isolation table in a sound-insulated and anechoic booth (Gretch-Ken, OR). Mice were attached to an EEG cable via the implanted post under brief anesthesia with isoflurane. The EEG recording set-up has been previously reported [51, 63]. Briefly, the attached cable was connected via a commutator to a TDT (Tucker Davis Technologies, FL) RA4LI/RA4PA headstage/pre-amp, which was connected to a TDT RZ6 multi-I/O processor. OpenEx (TDT) was used to simultaneously record EEG signals and operate the LED light used to synchronize the video and waveform data. TTL pulses were utilized to mark stimulus onsets on a separate channel in the collected EEG data. The EEG signals were recorded at a sampling rate of 24.414 kHz and down-sampled to 1024 Hz for analysis. All raw EEG recordings were visually examined prior to analysis for artifacts, including loss of signal or signs of clipping, but none were seen. Therefore, no EEG data was rejected. Sound evoked EEGs were recorded as follows.

#### Auditory ERP

After a 25 min habituation to the recording arena with no stimuli, narrowband noise pulses (6–12 kHz) were presented at 75 dB SPL (120 repetitions, 100 ms duration, 5 ms rise/fall time, 0.25 Hz repetition rate) using a speaker (MF1, Tucker Davis Technologies, FL) situated 20 cm above the floor of the arena. ERP analysis and statistics have been previously described [51, 63]. Briefly, the EEG trace was split into trials, using the TTL pulses to mark sound onset. Each trial was baseline corrected, such that the mean of the 250 ms baseline period prior to sound onset was subtracted from the trial trace for each trial. Each trial was then detrended (MATLAB detrend function) and all trials were averaged together. Time–frequency analysis was performed with a dynamic complex Morlet wavelet transform with Gabor normalization. The wavelet parameter was set for each frequency to optimize time–frequency resolution. The non-baseline normalized single trial power (STP) does not correct for mean baseline power levels, allowing for the identification of ongoing ‘background activity’ during stimulus presentation. To compare the responses across genotype at each developmental time point, a non-parametric permutation test was used, to find clusters of significant values [65]. First, a t-test was run on each time–frequency point for the two groups being compared, yielding the T-values for all points. T-values corresponding to *p* < 0.025 were considered significant. Clusters of significant T-values were found and their area was measured. Next, the group assignments were shuffled randomly, and the t-tests and cluster-measurements were run again on the surrogate groups. This surrogate analysis was performed 2000 times to generate a distribution of cluster sizes that we would expect to find by chance. Originally identified clusters that were larger than 95% of the surrogate clusters were considered significant. This method allows for the identification of significant differences between groups without performing excessive comparisons.

##### Gap-ASSR

The stimulus used to assess auditory temporal processing is termed the’40 Hz gaps-in-noise ASSR’ (auditory steady state response, henceforth, ‘gap-ASSR’) [51]. The stimulus contains alternating 250 ms segments of noise and gap interrupted noise presented at 75 dB SPL. The gaps are strategically placed 25 ms apart, resulting in a presentation rate of 40 Hz, a rate that produces the strongest ASSR signal when measured from the AC and frontal regions and may reflect the resonance frequency of the underlying neural circuits [66–72]. For each gap in noise segment, the gap width and modulation depth are chosen at random. Gaps of 2, 4, 6, 8, 10, or 12 ms widths and modulation depths of 75 and 100% were used. To measure the ability of the cortex to consistently respond to the gaps in noise, inter-trial phase clustering (ITPC) at 40 Hz was measured [73]. The EEG trace was transformed using a dynamic complex Morlet wavelet transform. The trials corresponding to each parametric pair (gap duration + modulation depth) were grouped together. The ITPC was calculated for each time–frequency point as the average vector for each of the phase unit vectors recorded across trials (trial count > 100 trials per parametric pair). The ITPC values at 40 Hz were averaged to extract the mean ITPC for the parametric pairs in the AC and FC.

### Statistics

Statistics were performed on GraphPad Prism (ERP) or R (gap-ASSR). To evaluate the effects of genotype (2 levels) and age (3 levels), two-way ANOVA was used for ERP analysis. Post hoc comparisons were carried out with Tukey’s and Bonferroni’s multiple comparisons test. The ERP data was tested for normality using Shapiro–Wilk test. A three-way repeated measures ANOVA was used for gap-ASSR analysis, with the three factors being genotype (2 levels) X age (3 levels) X gap duration (6 levels). A repeated measures ANOVA was chosen as multiple gap duration data points were collected from a single mouse in a recording session. Mauchly Tests for Sphericity were utilized and corrected for using the Greenhouse–Geisser corrections if necessary. Post hoc contrasts with Sidak corrections for multiple comparisons were used. Cortical regions (AC, FC) and modulation depths (75%, 100%) were analyzed separately. We evaluated the appropriateness of the data for analysis via ANOVA, in particular the assumption of the normality of the residuals. None of the residuals had measures of skewness or kurtosis that exceeded ± 2, which is one indication of acceptable normality [74]. Moreover, the residuals were evaluated via quantile–quantile plots. In each of the analyses, the correspondence between the theoretical normal distribution and the obtained residuals was within acceptable bounds.

## Results

The main goal of this study was to compare the developmental trajectory of auditory temporal processing and ERPs in WT and *Fmr1* KO mouse auditory and frontal cortex. We predicted that *Fmr1* KO mice would show a deficit in phase locking to rapid gaps in noise and larger ERP amplitudes compared to WT mice across all 3 ages in both AC and FC as markers of temporal processing and hypersensitivity phenotypes, respectively, in FXS.

### Abnormal temporal processing is seen in the FC, but not AC, during development

Auditory temporal processing was assessed using a 40 Hz gap-in-noise ASSR stimulus to probe the limits of the auditory and frontal cortices’ ability to consistently respond to brief gaps in noise. Decreasing the duration and modulation depth of the gaps reduces the likelihood of consistent response from the cortex, allowing for the detection of deviations between WT and KO mice responses and to track developmental changes. Both AC and FC in mice and humans produce robust 40 Hz ITPC to this type of stimulus, but how the response develops is not known in either species, nor is it known if there is a deficit in FXS [51, 72, 75].

**Fig. 1:**
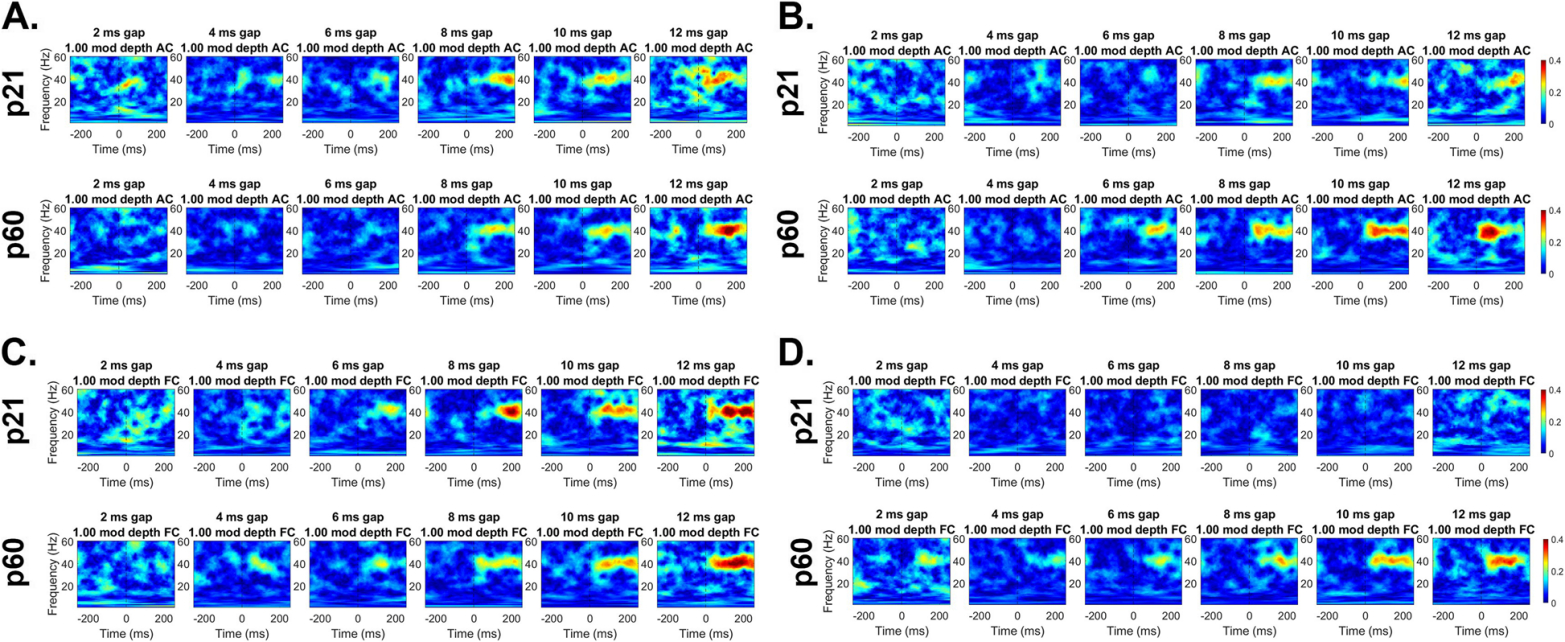
Abnormal auditory temporal processing during development in the *Fmr1* KO mice. Individual example heatmaps of ITPC generated at 40 Hz at multiple gap durations in p21 and p60 WT (A: AC, C: FC) and *Fmr1* KO (B: AC, D: FC) mice. Qualitative observations of these examples show deficits in cortical temporal processing at p21, but not at p60, in the KO mice. All panels show 100% modulation depth. The onset of the gap-ASSR stimulus is at 0 ms in each panel

**Fig. 2:**
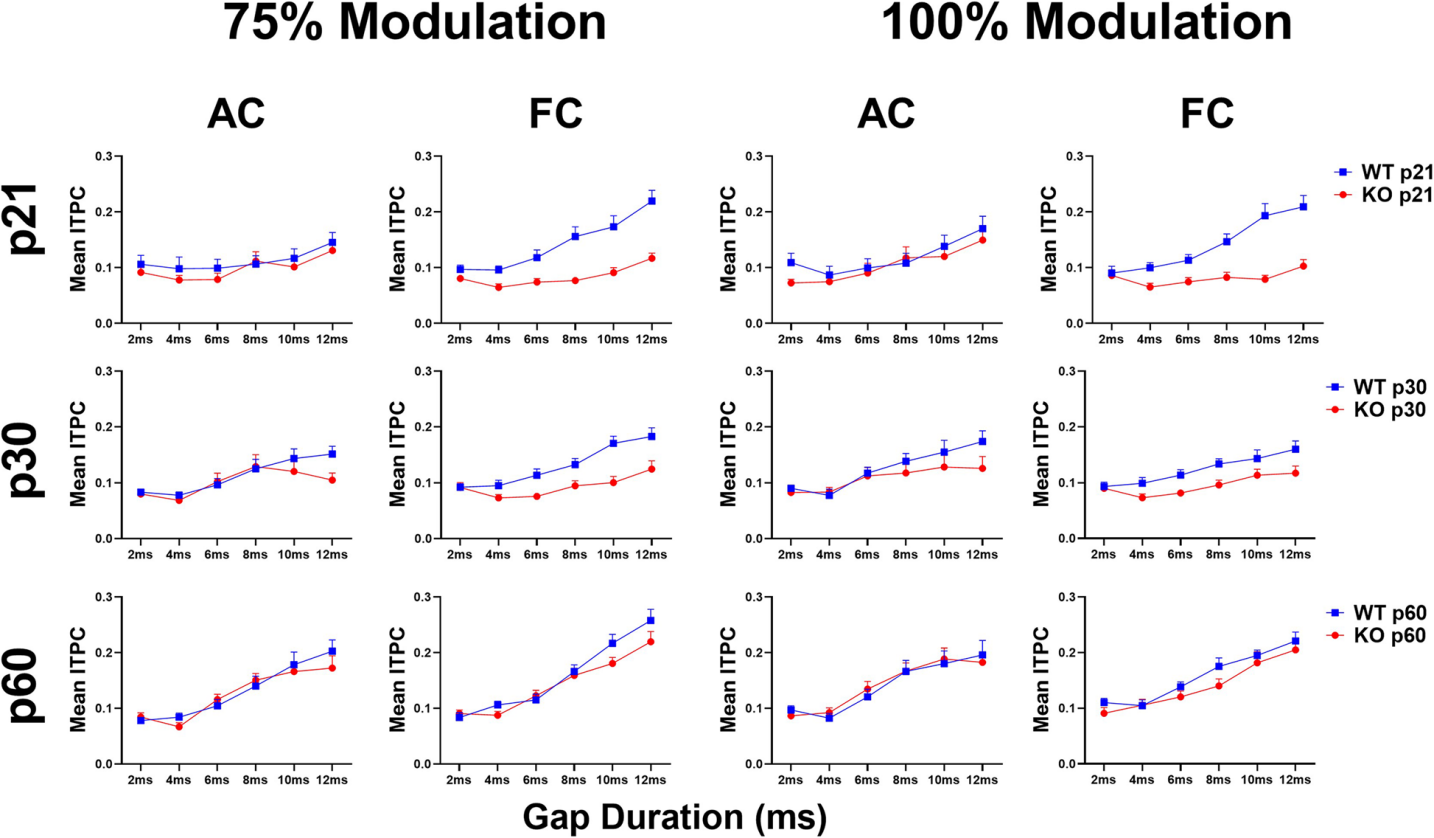
Population analysis shows temporal processing deficits in the FC during development in *Fmr1* KO mice. Each plot represents the group average ITPC values. Each row represents a different age group: p21 (top), p30 (middle), and p60 (bottom). The left columns represent AC and FC data at 75% modulation depth and the right columns represent AC and FC data at 100% modulation depth. ITPC increases with gap widths in both genotypes, as expected. *Fmr1* KO mice show significant deficits in the FC, but not the AC, at p21 and p30. Full data analysis is shown in Table 1

Figure 1 shows gap-ASSR heat maps of ITPC in example WT (Fig. 1A, C) and *Fmr1* KO (Fig. 1B, D) mice. In the AC, at p21, or in both AC and FC at p60, there are no clear qualitative differences in the ITPC. However, deficits are clearly seen in the FC at p21, with the KO ITPC barely emerging above background at 40 Hz. Table 1 and Fig. 2 shows the results of full statistical analyses using gap duration, age and genotype as factors.

**Table 1:** Full statistical analysis of gap-ASSR data

Cortical Region	Modulation Depth	Factor/Interaction	ANOVA Results	Adjusted *p*-value
AC	100%	Genotype	F(1,56) = 0.8631	0.3568
		Age	F(2,56) = 2.5463	0.0874
		**Gap Duration**	**F(5,280) = 24.0444**	** < 0.0001**
		Genotype x Gap Duration	F(5,280) = 1.5849	0.1986
		Age x Gap Duration	F(10,280) = 1.7898	0.1106
		Genotype x Age	F(2,56) = 0.3026	0.7401
		Genotype x Age x Gap Duration	F(10,280) = 0.6989	0.6392
AC	75%	Genotype	F(1,56) = 1.1929	0.2794
		Age	F(2,56) = 1.7843	0.1773
		**Gap Duration**	**F(5,280) = 21.2106**	** < 0.0001**
		Genotype x Gap Duration	F(5,280) = 2.5458	0.0571
		**Age x Gap Duration**	**F(10,280) = 3.2468**	**0.0046**
		Genotype x Age	F(2,56) = 0.0719	0.9307
		Genotype x Age x Gap Duration	F(10,280) = 0.5839	0.7444
FC	100%	**Genotype**	**F(1,56) = 23.7897**	** < 0.0001**
		**Age**	**F(2,56) = 12.3904**	** < 0.0001**
		**Gap Duration**	**F(5,280) = 20.6491**	** < 0.0001**
		**Genotype x Gap Duration**	**F(5,280) = 6.6448**	** < 0.0001**
		**Age x Gap Duration**	**F(10,280) = 5.3246**	** < 0.0001**
		**Genotype x Age**	**F(2,56) = 3.2364**	**0.0467**
		**Genotype x Age x Gap Duration**	**F(10,280) = 3.5686**	**0.00096**
FC	75%	**Genotype**	**F(1,56) = 31.7872**	** < 0.0001**
		**Age**	**F(2,56) = 16.0560**	** < 0.0001**
		**Gap Duration**	**F(5,280) = 29.1751**	** < 0.0001**
		**Genotype x Gap Duration**	**F(5,280) = 10.6887**	** < 0.0001**
		**Age x Gap Duration**	**F(10,280) = 5.7920**	** < 0.0001**
		**Genotype x Age**	**F(2,56) = 3.9569**	**0.0247**
		Genotype x Age x Gap Duration	F(10,280) = 1.0704	0.3839

Three-way repeated measures ANOVA results for gap-ASSR analysis. Mauchly Tests for Sphericity were utilized and p-values were corrected for multiple comparisons using the Greenhouse–Geisser corrections if necessary. See text for post hoc results. Bold text indicates statistical significance (*p* = or < 0.05)

### Auditory cortex

Gap-ASSR ITPC is significantly impacted by gap duration in the AC at both modulation depths. This is expected because it is easier for neural generators to phase lock responses to long gaps compared to short. There was no main effect of age in the AC at either 75% or 100% modulation, but there is an interaction of gap duration x age at 75%, suggesting that ITPC improves with age for longer gaps. Importantly, the genotype comparisons were not significant at any age or modulation depth in the AC (Fig. 2, 75% modulation – p21: *p* = 0.9223, p30: *p* = 0.9568, p60: *p* = 1.000; 100% modulation – p21: *p* = 0.8664, p30: *p* = 0.6906, p60: *p* = 1.000). Taken together, these data suggest developmental improvement in temporal processing, but no effects of the loss of FMRP, in the auditory cortex at any age.

### Frontal cortex

Similar to the AC, the frontal cortex showed main effects of gap duration, as expected. However, in contrast to the AC, FC gap-ASSR showed main effects of both age and genotype (Fig. 2) and a number of relevant interactions (Table 1). At both modulation depths, FC responses showed improvement with age indicating a strong developmental regulation of temporal processing in this region. At both modulation depths, *Fmr1* KO neurons showed significant deficits in ITPC compared to WT mice. The genotype X age interactions suggest a delay in ITPC development with adult FC showing no significant deficits. These results indicate a significant delay in the development of temporal processing in the *Fmr1* KO mice.

**Fig. 3:**
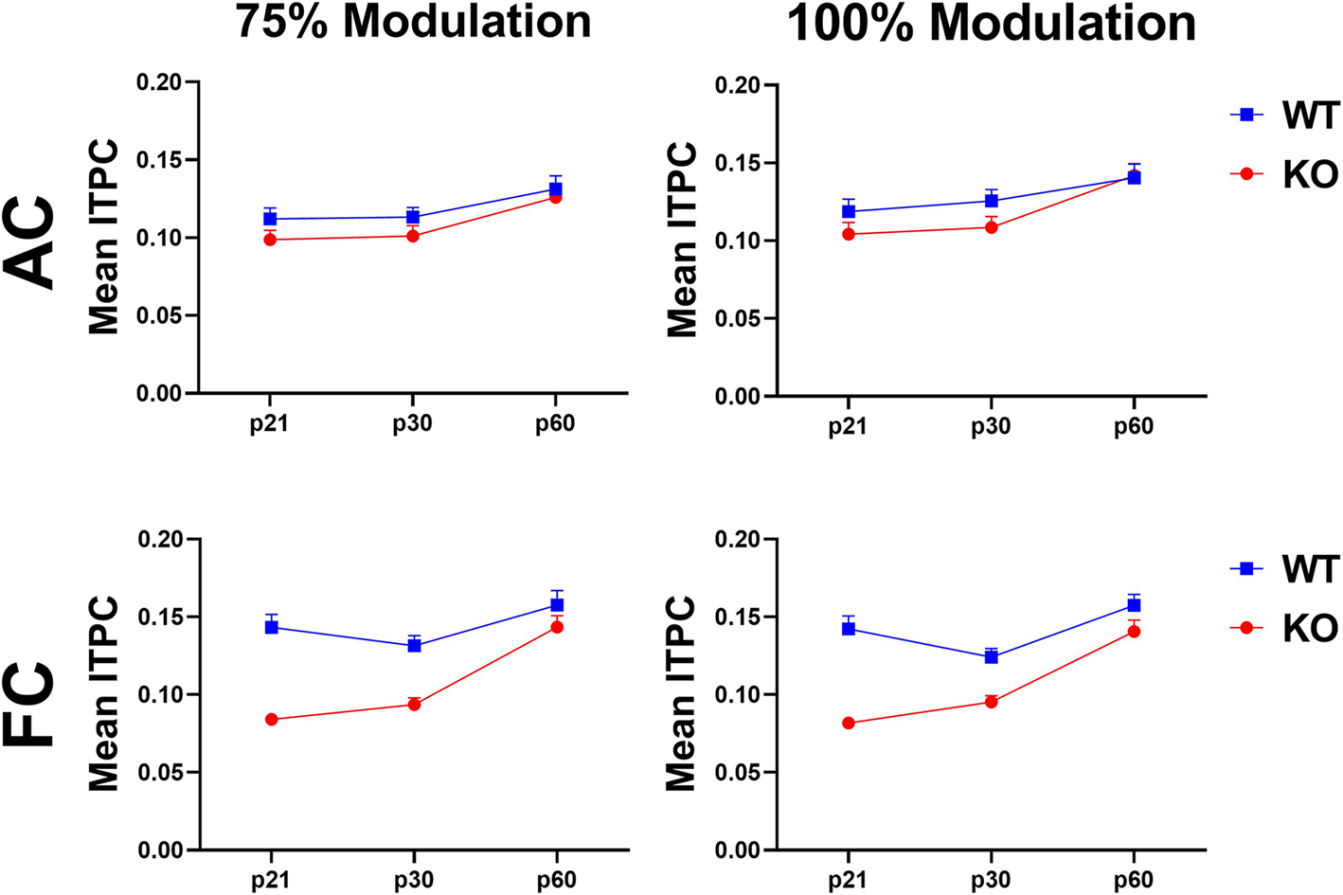
Auditory temporal processing improves with age in AC and FC, with a developmental delay in the FC. Each plot represents the group average ITPC values collapsed across gap widths. Columns represent different modulation depths and rows represent different cortical regions (Columns – left = 75% modulation, right = 100% modulation; Rows – top = AC, bottom = FC). KO mice show a significant ITPC deficit at p21 and p30 in the FC at both modulation depths, but not at p60. A genotype difference was not seen at any age or modulation depth in the AC

Evidence of a developmental delay in the FC is shown more directly by collapsing across gaps (Fig. 3). When collapsed across gap durations, KO mice show a significant ITPC deficit at p21 and p30 in the FC at both modulation depths that is not seen at p60 (75% modulation – p21: *p* < 0.0001, p30: *p* = 0.0022, p60: *p* = 0.8372; 100% modulation – p21: *p* < 0.0001, p30: *p* = 0.0548, p60: *p* = 0.6410). Taken together, these data show improvement in phase locking to gap-ASSR stimuli with development in both AC and FC, and an FC-specific delay in temporal processing in *Fmr1* KO mice.

### Development of gap-ASSR single trial power phenotypes in the *Fmr1* KO mice

**Fig. 4:**
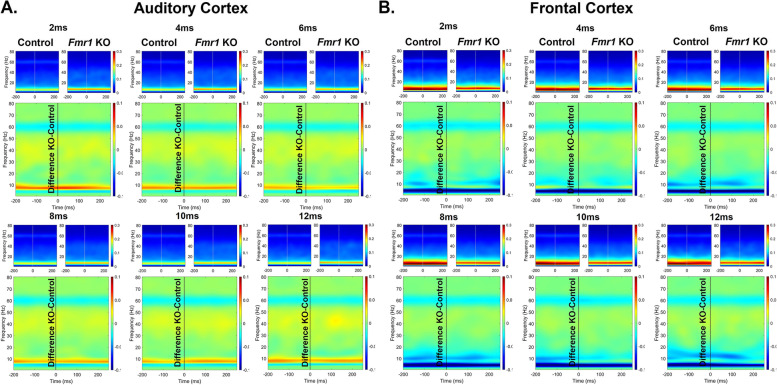
No genotype difference in single trial power (STP) of p21 mice during gap-ASSR stimulation. The heatmaps show non-baseline corrected normalized power, where red hues represent increased ongoing background activity, and blue hues represent a decrease. The smaller panels show group average STP at each gap width in WT and *Fmr1* KO mice. The larger panels show the difference between KO and WT. No significant differences were found in STP during the gap-ASSR stimulus in (A) AC or (B) FC at p21

**Fig. 5:**
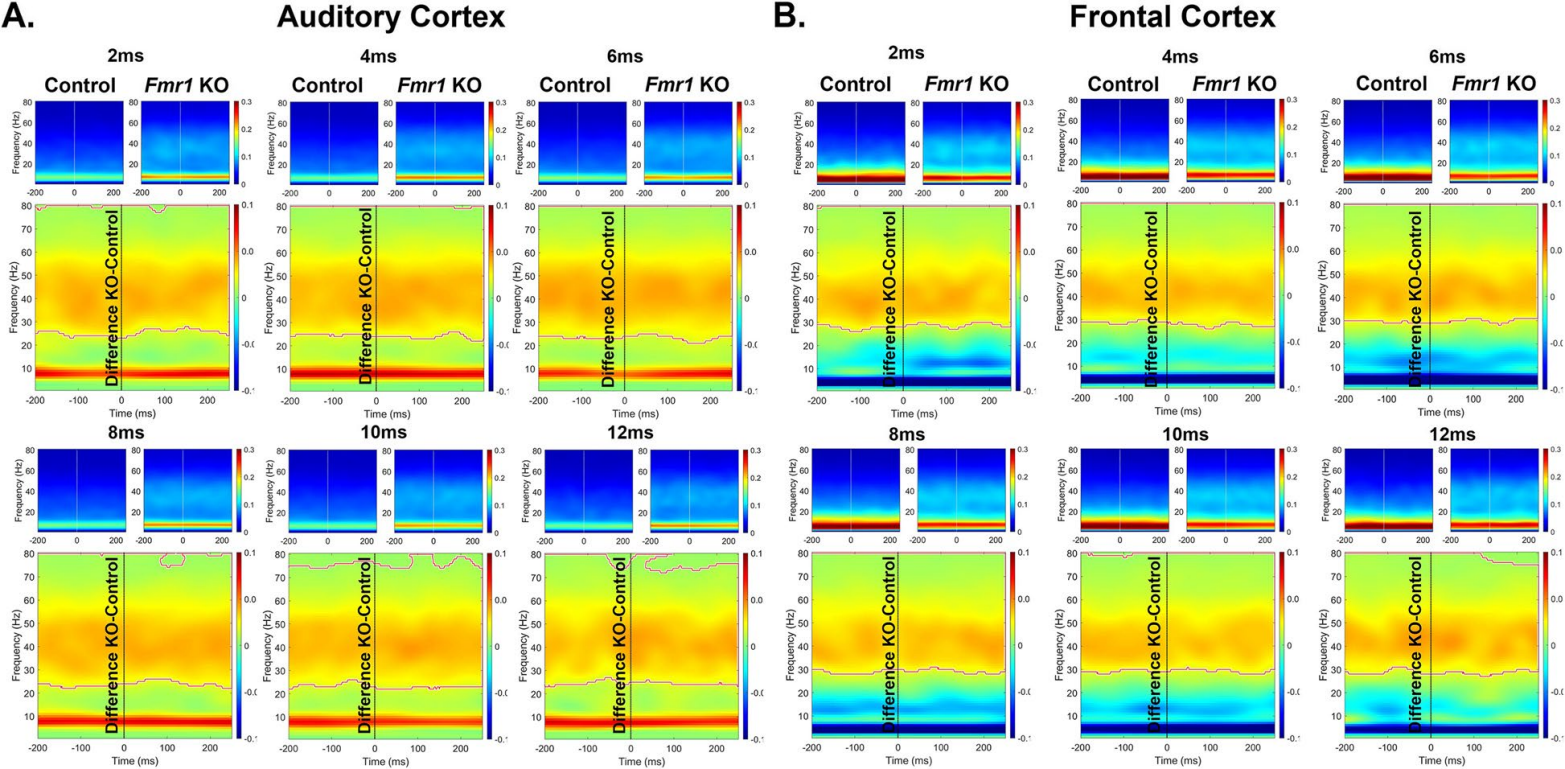
Significant elevation of STP in p30 *Fmr1* KO mice during gap-ASSR stimulation. The format of this figure is identical to that of Fig. 4. Significant differences between genotypes were found using a non-parametric permutation testing approach (see methods). Outlined regions (typically between 25–80 Hz) indicate clusters which are significantly different between WT and KO. *Fmr1* KO mice have increased gamma STP during the gap-ASSR stimulus in (**A**) AC and (**B**) FC at p30

**Fig. 6:**
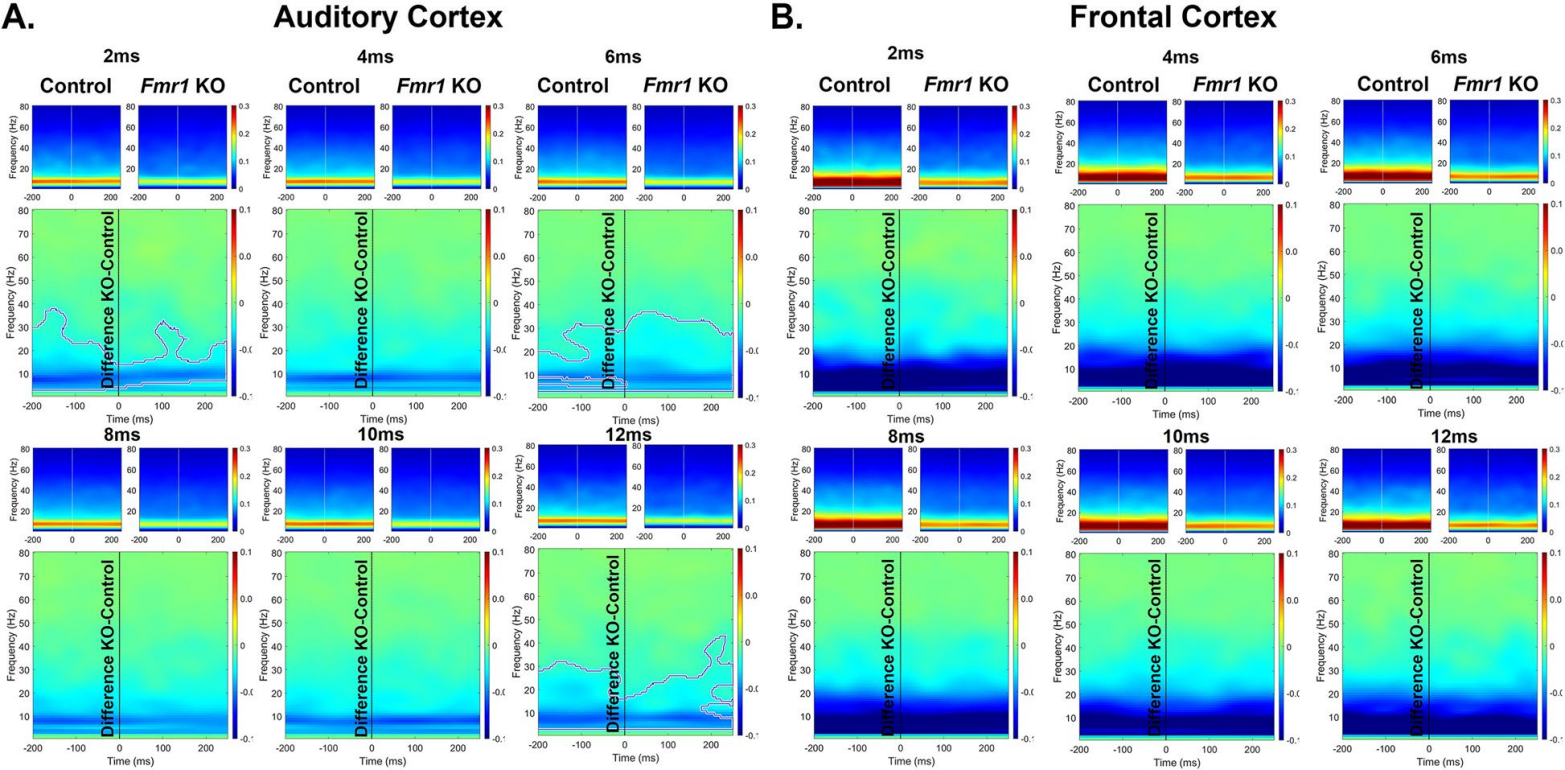
No difference or decreased STP in p60 *Fmr1* KO mice during gap-ASSR stimulation. The format of this figure is identical to that of Fig. 4 and 5. Outlined regions (typically < 30 Hz) indicate clusters which are significantly different between WT and KO. *Fmr1* KO mice show decreased STP at some gaps during the gap-ASSR stimulus in (**A**) AC but show no significant clusters in (**B**) FC at p60. Unlike at p30, there was no increase in STP in KO mice

It is possible that the reduced ITPC in developing *Fmr1* KO mouse FC arises from stimulus induced increases in non-phase locked activity (background noise) as suggested in humans with FXS [44, 45]. Therefore, we examined the single trial power (STP) during gap-ASSR stimulation across development and genotypes (Figs. 4, 5 and 6). At p21, there was no difference in STP across any of the gaps or cortical regions (Fig. 4). However, at p30, there was a significant elevation of STP in the KO, compared to the WT mice, and this was seen in both cortical regions (Fig. 5). The elevation in STP affected gamma band frequencies (25–80 Hz), with no differences in lower frequencies. In the adult group, the direction of STP differences was reversed in the AC, such that the *Fmr1* KO mice showed reduced STP, significantly affecting frequencies < 25 Hz. However, there were no STP differences in the FC. These data provide evidence for fluctuating single trial power gap-ASSR phenotypes through development. The lack of concurrence between the STP deficits and the gap-ASSR deficits across both cortical region and age indicates that the temporal processing deficit is not due to sound-induced increases in ongoing background activity.

### *Fmr1* KO mice show enhanced ERP amplitude in the AC and FC across development

**Fig. 7:**
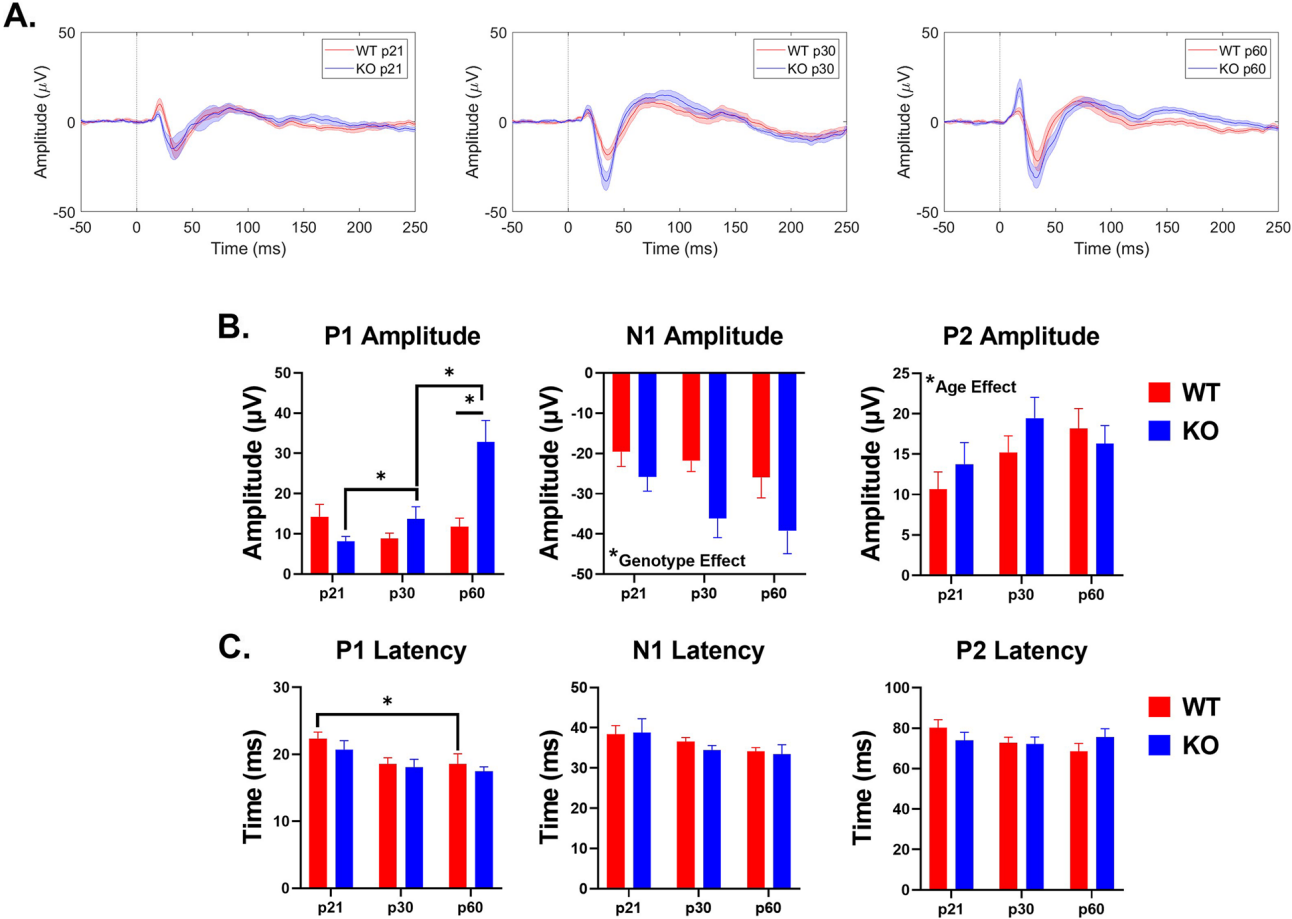
Age and genotype impact ERP amplitudes and latencies in the AC. **A** Average ERPs recorded in the AC for WT and KO mice at p21 (left), p30 (middle), and p60 (right). **B** Population averages of AC ERP wave amplitudes. P1 amplitude significantly increases in KO mice with development, but not WT mice. Adult KO mice have increased P1 amplitudes compared to WT. Genotype impacts N1 amplitudes. P2 amplitude are affected by age, but not genotype. **C** AC ERP wave latencies. P1 latency decreases with age in WT mice. Full analysis is shown in Table 2

**Fig. 8:**
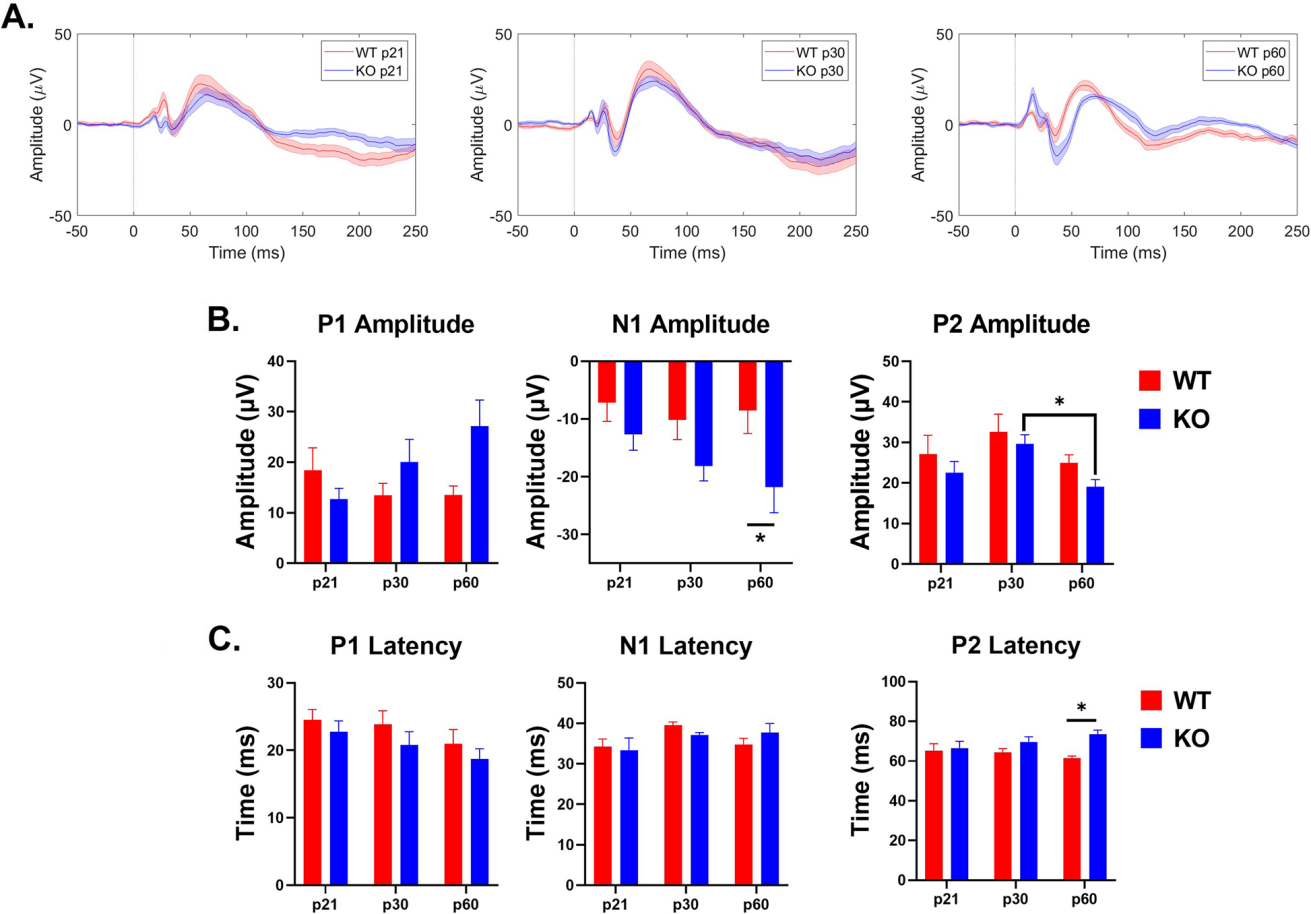
Age and genotype impact ERP amplitudes and latencies in the FC. **A** Average ERPs recorded from the FC for WT and KO mice at p21 (left), p30 (middle), and p60 (right). **B** FC ERP wave amplitudes. KO mice show a significant increase in P1 amplitude with development. N1 amplitudes are increased in adult KO mice. P2 amplitudes decrease with age in KO mice. **C** FC ERP wave latencies. P2 latency is increased in adult KO mice. Full analysis is shown in Table 2

ERPs consist of a series of voltage fluctuations, referred to as ‘waves’ (P1, N1, P2), which are evoked at specific latencies after sound onset. Each of the waveforms are associated with the population activity in specific brain regions. Measuring the amplitudes and latencies of these waves allow for the assessment of response synchrony or hypersensitivity to sound presentation. We also characterized non-baseline normalized STP in response to narrowband noise bursts as abnormal power has been identified in humans with FXS during auditory stimulus presentation [44, 45]. Table 2 and Figs. 7 and 8 show the complete ANOVA analyses of ERP data across development and genotypes. The major results in the two cortical regions are highlighted below.

**Table 2:** Full statistical analysis of ERP data

Cortical Region	ERP Component	Factor	ANOVA Results	*p*-value
AC	P1 Amplitude:	**Age**	**F(2,56) = 8.807**	**0.0005**
		**Genotype**	**F(1,56) = 6.863**	**0.0113**
		**Age x Genotype**	**F(2,56) = 9.972**	**0.0002**
	N1 Amplitude:	Age	F(2,56) = 2.565	0.0859
		**Genotype**	**F(1,56) = 9.680**	**0.0029**
		Age x Genotype	F(2,56) = 0.4776	0.6228
	P2 Amplitude:	**Age**	**F(2,56) = 3.007**	**0.0575**
		Genotype	F(1,56) = 0.8885	0.3499
		Age x Genotype	F(2,56) = 0.9607	0.3888
FC	P1 Amplitude:	Age	F(2,56) = 0.9323	0.3997
		Genotype	F(1,56) = 2.582	0.1137
		**Age x Genotype**	**F(2,56) = 3.595**	**0.0340**
	N1 Amplitude:	Age	F(2,56) = 1.229	0.3002
		**Genotype**	**F(1,56) = 9.559**	**0.0031**
		Age x Genotype	F(2,56) = 0.6309	0.5359
	P2 Amplitude:	**Age**	**F(2,56) = 4.461**	**0.0159**
		Genotype	F(1,56) = 3.105	0.0835
		Age x Genotype	F(2,56) = 0.1059	0.8997
AC	P1 Latency:	**Age**	**F(2,56) = 5.764**	**0.0053**
		Genotype	F(1,56) = 1.320	0.2555
		Age x Genotype	F(2,56) = 0.1276	0.8804
	N1 Latency:	Age	F(2,56) = 2.884	0.0643
		Genotype	F(1,56) = 0.2235	0.6382
		Age x Genotype	F(2,56) = 0.1730	0.8416
	P2 Latency:	Age	F(2,56) = 1.095	0.3417
		Genotype	F(1,56) = 6.018e-005	0.9938
		Age x Genotype	F(2,56) = 1.667	0.1981
FC	P1 Latency:	Age	F(2,56) = 2.283	0.1114
		Genotype	F(1,56) = 2.459	0.1225
		Age x Genotype	F(2,56) = 0.0603	0.9415
	N1 Latency:	Age	F(2,56) = 2.809	0.0688
		Genotype	F(1,56) = 0.0073	0.9323
		Age x Genotype	F(2,56) = 1.123	0.3325
	P2 Latency:	Age	F(2,56) = 0.1817	0.8343
		**Genotype**	**F(1,56) = 8.761**	**0.0045**
		Age x Genotype	F(2,56) = 2.412	0.0989

Two-way ANOVA results for ERP analysis. Post hoc comparisons were done using Tukey’s and Bonferroni’s multiple comparisons tests and p-values were adjusted accordingly. See text for post hoc results. Bold text indicates statistical significance (*p* = or < 0.05)

### Auditory cortex

ERP P1 amplitude in the AC significantly increases in *Fmr1* KO mice with development (interaction effect: *p* = 0.0002; main effect of age: *p* = 0.0005; KO p21-p60: *p* < 0.0001; KO p30-p60: *p* = 0.0001). These mice also have significantly higher P1 amplitudes compared to WT at p60 (main effect of genotype: *p* = 0.0113; post hoc: *p* < 0.0001). We found a main effect of genotype on N1 amplitudes. Additionally, we report a significant main effect of age on P2 amplitude (*p* = 0.0575). P1 latencies are impacted by age specifically in WT mice, with latencies decreasing with age (main effect of age: *p* = 0.0053; WT p21-p60: *p* = 0.0537). These data show increased ERP amplitudes in the AC of *Fmr1* KO mice as observed consistently in humans with FXS, but indicate early emergence of hypersensitivity.

### Frontal cortex

Similar to the AC, *Fmr1* KO mice show a significant increase in P1 amplitude with development in the FC (interaction effect: *p* = 0.034). N1 amplitudes were increased significantly in adult *Fmr1* KO mice (main effect of genotype: *p* = 0.0031; WT-KO p60: *p* = 0.0251). Additionally, P2 amplitudes decrease with age in KO mice (main effect of age: *p* = 0.0159; KO p30-p60: *p* = 0.0510). P2 latency was slower in adult *Fmr1* KO mice (main effect of age: *p* = 0.0045; WT-KO p60: *p* = 0.0030). These data indicate that *Fmr1* KO mice have abnormally elevated N1/P1 ERP amplitudes in the frontal cortex.

### Development of ERP single trial power phenotypes in the *Fmr1* KO mice

**Fig. 9:**
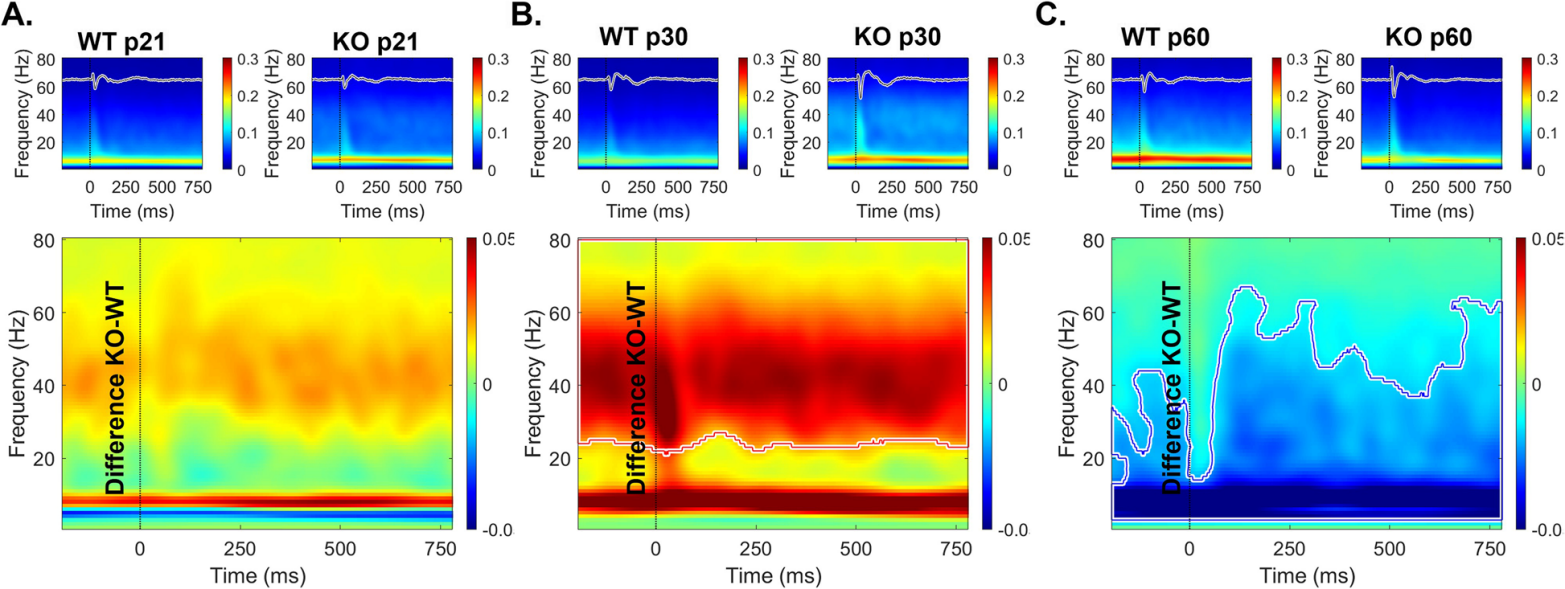
Non-baseline normalized STP during ERP stimulation is altered in *Fmr1* KO in the AC during development. The format is similar to Figs. 4–6, except these are obtained during ERP stimulation. Outlined regions indicate clusters which are significantly different between WT and KO. **A** Young *Fmr1* KO mice show no difference in STP at p21. **B** KO mice have increased background activity in the gamma range at p30. **C** Adult KO mice show decreased STP in the beta and gamma frequency ranges compared to WT

**Fig. 10:**
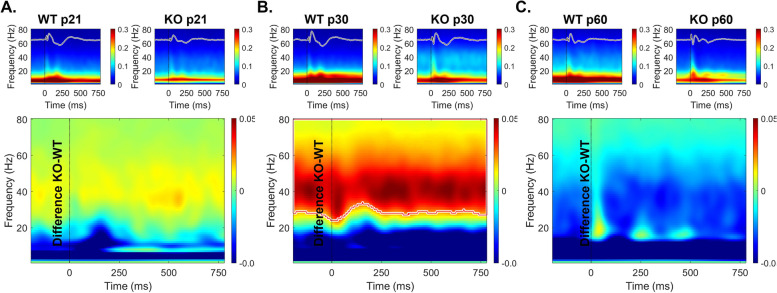
Non-baseline normalized STP during ERP stimulation is altered in *Fmr1* KO in the FC during development. Figure format is the same as in Fig. 4–6. **A** Young *Fmr1* KO mice show no difference in STP at p21. **B** KO mice have increased background activity in the gamma frequencies at p30. **C** Adult *Fmr1* KO mice show no significant difference in STP at p60

In addition to ERP peak amplitude and latency, we analyzed STP during the stimulus train used for ERP measurement (Figs. 9, 10). The STP phenotypes were similar to those found with the gap-ASSR paradigm. There was no genotype difference in STP at p21 in either AC (Fig. 9) or FC (Fig. 10). At p30, KO mice showed elevated STP in both AC and FC, with effects limited to frequencies between 25–80 Hz. At p60, the KO mouse AC showed reduced STP at frequencies below 60 Hz, but there was no difference in the FC. These results support the idea of developmental fluctuations in background power phenotypes in FXS.

## Discussion

The major and novel contribution of this study is the identification of developmental trajectories of auditory temporal processing in two cortical regions of WT and *Fmr1* KO mice. We recorded 40 Hz gap-in-noise ASSR from the AC and FC at three different ages as a measure of temporal processing. We also quantified ERP amplitudes/latencies and sound evoked single trial power to determine if abnormally elevated EEG power is developmentally correlated with temporal processing deficits. The results show genotype, cortical region- and age-specific abnormalities in gap-ASSR responses and ERPs. Interestingly, significant developmental delay was seen in gap-ASSR responses in the FC, but not the AC, of *Fmr1* KO mice. ERP N1 amplitudes were larger across development in both AC and FC of the KO mouse. The non-phase locked STP phenotypes showed developmental fluctuations. Between p21 and p30 there was an increase in STP during both gap-ASSR and ERP recordings in the *Fmr1* KO mice, and at p60 there was a reversal of this phenotype. Taken together, these data provide novel evidence for abnormal development of temporal processing in the frontal cortex, and hypersensitive responses in both auditory and frontal cortex in the *Fmr1* KO mouse model of FXS. The data do not support the notion that hypersensitive cortical responses underlie temporal processing deficits in developing *Fmr1* KO mice as there was no developmental correlation between the two measures. These phenotypes may arise from independent mechanisms. The robust developmental delays in gap-ASSR EEG responses in KO mice provide physiological tools to evaluate underlying mechanisms and identify treatment targets and windows.

The WT mouse shows significant developmental improvement in gap-ASSR EEG responses, providing a reference for mouse models of other ASD and neurodevelopmental disorders. This is consistent with findings in the rat auditory cortex in which the percentage of neurons with short neural gap detection thresholds increases from juveniles to adults [76]. These neural improvements in gap processing may underlie perceptual improvement in gap detection thresholds in children, a factor that is correlated with improved language in development [6, 77–80].

Expressive and receptive language deficits are consistently reported in FXS, but the underlying mechanisms are unclear [81]. Children with FXS express developmental delays in multiple cognitive categories necessary for language maturation, such as auditory short-term memory and attention [82–87]. In addition to these cognitive factors, delayed temporal processing and auditory hypersensitivity may underlie speech and language delays in FXS [4, 5]. In developmental disorders and in aging, gap processing has been used to analyze auditory temporal acuity across groups [51, 63, 88, 89]. Increased gap-detection thresholds are seen in children with ASD and impaired gap detection thresholds in children correlate with lower phonological scores [8]. The 40 Hz gap-in-noise-ASSR paradigm used here tests the ability of neural generators of EEGs in the AC and FC to phase lock consistently across trials and can be used in humans with FXS to determine if similar cortical region-specific temporal processing deficits are present in patients. By varying the modulation depth and gap widths, it is possible to compare temporal processing acuity of auditory systems across groups [52, 53]. The cortical mechanisms of gap processing are also beginning to be understood [90, 91]. Future EEG studies in children with FXS should examine if temporal gap processing deficits are present early in development, and if they are related to development of language abilities. This may provide the basis for adaptive training of children with rapidly changing stimuli, including gaps, to improve speech recognition and language [92].

The current study focused on 40 Hz ASSR for multiple reasons. Gamma band deficits have been consistently observed in humans with FXS and *Fmr1* KO mice across strains and ages [31, 44, 45, 47–49]. There is also a developmental delay in the maturation of parvalbumin-expressing inhibitory neurons and the perineuronal nets that surround them [59]. As these neurons are involved in generating gamma band oscillations, we predicted 40 Hz ASSR deficits. Another reason for focusing on the 40 Hz ASSR is that the auditory cortex has a resonance at that frequency, and therefore, produces the largest power in EEG responses at 40 Hz [72, 93, 94]. In addition, the mechanisms of 40 Hz ASSR have been studied, including descriptions of topography across regions and the role of basal forebrain neurons [71, 95].

More relevant to speech processing, there is a strong link between gamma band oscillations and phoneme processing, with gamma oscillations parsing speech input in the phoneme range [96]. The slower oscillations (delta-theta) may be more relevant to aspects of intonation and syllabic rates, and other aspects of speech with slower evolution. Based on such observations, the ‘asymmetric sampling in time’ hypothesis for speech processing has been proposed in which gamma oscillations play a significant role in phoneme processing [96–98]. Gamma resolution parsing may provide sufficient cues in separating closely spaced inputs (e.g., voice onset time, formant transitions), facilitating speech recognition. Future studies measuring 10 and 20 Hz ASSRs in the *Fmr1* KO and WT mice will provide insights into the mechanisms of speech deficits in humans with FXS.

### ERP deficits in *Fmr1* KO mice

The P1-N1-P2 ERP complex marks the pre-attentive detection of sound and can vary with stimulus features. Consistent with a number of studies in humans with FXS, and our previous studies in adult and developing mice, ERP component amplitudes were higher in the *Fmr1* KO mice compared to WT mice [Humans: 86–91; Mice: 47–48,92]. We found N1 amplitude, which are generated from frontal and temporal lobes [99] and marks synchronous activity within the cortex, to be higher in both AC and FC of the KO mice. This is consistent with calcium imaging studies that showed abnormally high synchronous activity in the *Fmr1* KO mouse cortex, and may arise from abnormal activity of parvalbumin positive inhibitory interneurons [59, 100, 101]. Reduced habituation of responses in mice [102] and humans [38] may also contribute to larger N1 amplitudes because the reported amplitude is the average of responses to multiple trials. We also observed a main effect of genotype and/or genotype X age interactions for P1 amplitude, with KO mice showing larger amplitudes. P1 amplitudes mark thalamocortical input activity, suggesting enhanced input drive of the cortex in the KO mice. This may arise from reduced input layer 4 thalamocortical drive of fast-spiking (putative parvalbumin positive) inhibitory interneurons in the KO cortex as shown by Gibson et al. and Patel et al. [103, 104]. P2 amplitudes are thought to be related to arousal as auditory input to the mesencephalic reticular activating system contributes to P2 generation [105]. There was no genotype difference in the AC. In the FC, however, there was a trend towards decreased P2 amplitude in the KO mice, suggesting the potential for reduced arousal and attention during development. The enhanced evoked responses and reduced habituation in FXS may lead to reduced ability for auditory change detection as shown by Van der Molen et al. [106]. Such sensory discrimination deficits may lead to speech and language abnormalities in FXS. A recent study in humans demonstrated a link between habituation and language abilities in children with FXS [107]. Specifically, it was shown that weaker P1 responses to later stimuli in a habituation train as well as larger habituation of P1 was associated with increased receptive and expressive language abilities, suggesting that habituation to repeated tones impacts language abilities in children with FXS.

### Enhanced gamma band power in background activity in *Fmr1* KO mice

The single trial power (STP) allows for the identification of ongoing ‘background activity’ during stimulus presentation as it does not correct for mean baseline power levels. It has been suggested that this non-phase locked power reflects relatively slow integrative processes that may impact stimulus or response processing [108]. These processes include top-down and sustained attention, decision-making, and perceptual inference, and are suggested to result from intrinsic network interactions rather than external stimuli [109, 110]. Our results show developmental fluctuations in STP phenotypes in *Fmr1* KO mice, with adolescent KO mice (p30) having increased STP during ERP and gap-ASSR stimuli in the AC and FC compared to WT mice. The increase in STP was seen in the gamma band (30–80 Hz), consistent with data from Ethridge et al. from humans with FXS compared to typically developing control [45]. Human data, recorded from adolescents and young adults, also shows elevated gamma band STP across multiple stimulus types. Importantly, the elevated gamma power showed correlations with IQ and distractibility. These data suggest increased on-going activity that may be a result of hyperactive network connections across species, and with potential clinical implications in humans. The reasons why the phenotypes fluctuate over development is unclear. It is also not known if similar age-effects are seen in humans with FXS. With neurodevelopmental disorders, it is sometimes difficult to disambiguate the direct effect of the mutation from the effect of potential compensatory (e.g., homeostatic) adjustments in activity levels.

A previous study (Wen et al., 2019) reported increased resting EEG gamma power in the frontal cortex of adult *Fmr1* KO mice (FVB strain, the same used here) [48]. We did not observe increased gamma power in the STP data in the present study. While both resting EEG and sound evoked STP can be considered as background activity, the differences across the two studies can be explained by how these measures are calculated. Resting EEGs are recorded in the absence of any sound stimuli, but the STP calculated is background during sound stimulation. The animal is likely in a different state of arousal in the presence of sounds compared to the resting condition leading to observed differences between the different measures of background gamma activity.

### Delayed development of temporal processing in *Fmr1* KO mouse frontal cortex

Perhaps the most surprising result of the study is that developmental delays in temporal processing were seen in the FC, but not the AC. These data suggest that FC does not simply inherit auditory responses from the AC, but that additional local processing within the FC and/or auditory pathways that bypass the AC may be involved in producing phase locked responses in the FC. Very little is known regarding mechanisms of auditory processing in the FC. Robust frontal cortex ASSR power is seen in both human [75] and mouse EEG recordings [72]. Indeed, topographical distribution of ASSR power and precision favors more frontal regions in both species. Kim et al. [71] and Hwang et al. [72] showed that optogenetic stimulation of GABAergic parvalbumin neurons in the mouse nucleus basalis preferentially increased frontal cortex 40 Hz ASSR oscillations. This suggests independent modulation of ASSR in the FC that may be abnormal in early development in FXS. The idea that FC can robustly mount ASSR, and independently show deficits, is supported by two other lines of evidence. Clark et al. showed in an autoimmune disorder mouse model that gap processing is affected in the FC, while remaining normal in the AC [57]. Wieczerzak et al. reported reduced recovery of ASSR in FC, rather than AC, following noise induced hearing loss [55]. The fact that gap-ASSR deficits are seen in early development in the FC, and the not the AC in *Fmr1* KO mice, suggests temporal processing may be abnormal across multiple sensory modalities in FXS. If a similar developmental regional difference in temporal processing is seen in humans with FXS, this would suggest speech processing and language function may be affected across multiple modalities [111].

An important consequence of abnormal temporal processing in the FC may be related to how FC-AC top down interactions function during development. FC induces top-down modulation of AC responses in a task- and attention-dependent fashion. Fritz et al. hypothesized that the FC modulates AC neuron receptive fields depending on the task and selective attention [112]. FC-AC connection and its modulation of speech have also been evaluated in humans with FXS. Speech production depends on feedforward control and the synchronization of neural oscillations between the FC and AC. Specifically, the interactions of these two regions allow for comparison of the corollary discharge of intended speech generated from an efference copy of speech to the actual speech sounds produced, a process essential for making adaptive adjustments to optimize future speech [113]. A study of humans utilizing a talk-listen paradigm found that in the time window prior to speech production, individuals with FXS have decreased pre-speech activity, including frontotemporal connectivity, as well as increased frontal gamma power compared to controls. These discrepancies brought about less intelligible speech and correlated with increased social communication deficits [113]. Abnormal functional connectivity between FC and AC is also suggested by Zhang et al., (2021) who showed reduced long-range connectivity in *Fmr1* KO mice [114]. Future studies will examine phase connectivity between FC and AC during different sound stimulation paradigms including the gap-ASSR. Taken together, the connections between the FC and AC are essential for shaping sensory responses and disruptions may cause speech and language impairments. A mismatched development pattern between these two regions in humans, as seen in the present study of mice, could possibly give rise to language abnormalities in FXS.

## Conclusions

We have identified a developmental delay in auditory temporal processing in the FXS model mouse. The p21-p30 window is a critical period of development in *Fmr1* KO mice that is marked by cortical hyperexcitability and reduced inhibitory interneuron function [48, 59, 115]. This delayed development is similar to other studies in *Fmr1* KO mice. For example, in the somatosensory cortex, *Fmr1* KO mice show delayed maturation of GABAergic inhibition and decreased synaptic connectivity that eventually normalize to WT levels in adults [116, 117]. Brain development is a precise process that is determined by accurately cued stages of gene expression, molecular guidance cues and intrinsic neuronal activity [118, 119]. The timing of these developmental stages, known as critical periods, is imperative for accurate neuronal migration, circuit formation and synaptic refinement [120]. Disruptions of critical period timelines cause long term impairments in behavioral phenotypes. Even though responses may be normalized in the adult, abnormal critical period development will have long-term consequences for behaviors that build on normal development of responses. For example, developmental delay in FC temporal processing may lead to long term abnormalities in behaviors that depend on accurate temporal processing such as speech, language and binaural processing. In order to effectively treat humans with FXS, it is imperative to understand the developmental trajectory of phenotypes that are likely to be used as clinical outcome measures, as opposed to just adult comparisons. Future studies should evaluate temporal processing across age to determine if similar delays in development are present in humans with FXS, and if the delay relates to language function.

## Data Availability

The data that support the findings of this study are available from the corresponding author with reasonable request.

## Abbreviations

ASD: Autism spectrum disorders
KO: Knock-out
FXS: Fragile X syndrome
WT: Wild-type
ERP: Event related potentials
ASSR: Auditory steady state response
p: Postnatal
*Fmr1*: Fragile X messenger ribonucleoprotein gene
FMRP: Fragile X messenger ribonucleoprotein
AC: Auditory cortex
FC: Frontal cortex
i.p.: Intraperitoneal
STP: Single trial power
ITPC: Inter-trial phase clustering
